# An Investigation of Loneliness and Perceived Social Support Among Single and Partnered Young Adults

**DOI:** 10.1007/s12144-015-9337-7

**Published:** 2015-06-11

**Authors:** Katarzyna Adamczyk

**Affiliations:** Institute of Psychology, Adam Mickiewicz University, ul. A. Szamarzewskiego 89/AB, 60-568 Poznań, Poland

**Keywords:** Loneliness, Perceived social support, Single, Romantic relationships, Young adults

## Abstract

This study investigated the possible differences between single individuals and individuals in nonmarital romantic relationships in the domains of emotional (romantic and family) and social loneliness, and of perceived social support from family, friends and significant others. Based on a Polish university-student sample of 315 participants (167 women and 148 men) aged 19 to 25 years (*M* = 21.90, *SD* = 2.15), single relationship status was related to greater romantic and family loneliness, and to less perceived social support from significant others and family. Women reported a lower level of social loneliness and a higher level of perceived social support in comparison to men. Relationship status interacted with gender in predicting perceived social support from significant others and friends. Finally, the duration of remaining single and significant others’ support were found to be predictive of single young adults’ romantic loneliness. In addition, perceived social support from family and significant others were found to moderate the relationship between the duration of remaining single and romantic loneliness. In particular, high family support and medium-high support from significant others mitigated the negative impact of being single for a long time on romantic loneliness.

## Introduction

Despite the significance attached to romantic relationships, often identified with marital relationships, and prevalent social expectation of having a romantic partner (DePaulo and Morris 2005), some people remain single for different reasons, which include, for example, personal choice, external circumstances, personal deficits or self-blame (Austrom and Hanel 1985; Frazier et al. 1996), or willingness to express one’s own individualization (i.e., individual autonomy and independence from traditions and institutions) (Poortman and Liefbroer 2010). In the past decades in Europe and the United States the number of single persons has risen, and this trend will probably continue in these regions (Poortman and Liefbroer 2010). The similar tendency is also observed in Poland (i.e., the country where the present study was conducted; Such-Pyrgiel 2014). The rise in singlehood is, in turn, related to the aging of society, postponement of union formation and the rising rate of divorce and separation (Poortman and Liefbroer 2010; Slany 2006). For example, recently in Poland there has been a trend to postpone marriage until 30 to 34 years of age, and a noticeable increase of single individuals (from 27.10 % men in 2002 to 32.80 % in 2011, and from 19.10 % women in 2002 to 23.90 % in 2011) (Sytuacja demograficzna Polski. Raport Rządowej Rady Ludnościowej 2012)

Although in Polish society, as in many societies around the world, singlehood is becoming more common among young adults because they postpone their decision to marry (e.g., Jones et al. 2012; Żurek 2008), singles are often the object of many stereotypes and prejudices (e.g., Greitemeyer 2009; Morris and Osburn 2015; Ochnik and Mandal 2015). For example, a commonly held societal belief is that loneliness is caused by a lack of a romantic partner and is cured by being in a romantic relationship (Seepersad et al. 2008). People assume that the never marrieds are lonely, isolated from their families, or that they do not have families (Greitemeyer 2009; DePaulo and Morris 2005; Keith 2003). At the same time, many single people live with their parents or friends, or they cohabitate with partners (Turner and Helms 1999), and they display a relatively good functioning (Keith 2003).

### Loneliness

In line with the attachment-cognitive approach loneliness is a result of an individual’s feeling a lack of strong, intimate bonds with significant others (Bowlby 1973; Weiss 1973). Loneliness may be conceptualized as a multifaceted and domain-specific phenomenon. Weiss (1973) was the first to describe loneliness as a multidimensional experience and proposed a distinction between social loneliness as a result of an inadequate access to social relationships such as a network of peers, co-workers, neighbours, or friends, and emotional loneliness perceived as a lack of close or intimate relationships which are characteristic of ties with a romantic partner, parent, or child. Emotional loneliness is primarily related to “the absence of a partner, that is, with the absence of an exclusive, close, and intimate tie” (Dykstra and Fokkema 2007, p. 9). In turn, social loneliness is related to a perceived deficiency in social networks, or a lack of social relations or social activities (Russell et al. 1984; Weiss 1973). Furthermore, on the basis of Weiss (1973) distinction between the experience of social isolation (social loneliness) and emotional isolation (emotional loneliness), DiTommaso and Spinner (1993, 1997) noted that emotional loneliness appeared to be comprised of two domains, that is, family emotional loneliness and romantic emotional loneliness.

The lack of romantic partners or intimate relationships may be an important perceived causal factor for one’s present feelings of loneliness (e.g., Rokach and Brock 1998). In particular, marriage is recognized to be a main factor which protects against loneliness for both married men and women who experience lower loneliness in comparison to non-married persons (Ayalon et al. 2013). Furthermore, results of a study on a broad range of participants aged 18 to 54 years showed that married individuals and individuals living with a significant other reported less romantic loneliness than those who were not in such relationships (Bernardon et al. 2011). DiTommaso and Spinner (1993) revealed that being involved in a romantic relationship was significantly related to lower levels of romantic loneliness, but was only weakly linked to family and social loneliness. Similarly, Çeçen (2007) found that being involved in a romantic relationship was related to lower scores on romantic loneliness, while not being involved in a romantic relationship was related to higher scores on the romantic scale but was not associated with scores on the family or social loneliness scales. Moreover, individuals who recently reported an ending of a romantic relationship reported higher romantic loneliness than those who did not (Wang et al. 2008).

Alongside the analysis of the link between relationship status and loneliness, an important issue concerns the linkage between romantic loneliness and the duration of remaining single. To the best of my knowledge, this issue has not yet been raised in prior studies. What may be of help when thinking of the linkage between romantic loneliness and the duration of remaining single is the temporal approach to loneliness (e.g., Beck and Young 1978). However, this approach distinguishes different types of loneliness in regard to differences in the scope of duration of loneliness, not in regard to the duration of a situation which may be recognized as associated with loneliness or as a cause of loneliness. For example, Beck and Young (1978) described the following three types of loneliness from the temporal perspective: chronic, situational, and transient loneliness. The first type of loneliness applies to situations when an individual has failed to establish satisfactory social relationships over years. The second type of loneliness is related to unexpected negative events such as the death of a loved one or major negative changes in one’s status (Beck and Young 1978). Finally, transient loneliness concerns momentary feelings of occasionally, but universally, experienced emptiness. This type of loneliness occurs, for example after leaving a meeting with a group (such as a reunion) (Wang et al. 2008). It is natural that individuals feel lonely at some point in their lives, and sometimes situational factors can increase the frequency or chronicity of loneliness (Cacioppo et al. 2006). One of these factors may be the situation of remaining single. For some people remaining single is merely a certain phase in life before getting married or being committed in a serious relationship, and for some people it is a prolonged state lasting against their will (Kaiser and Kashy 2005).

In regard to gender differences in the domain of loneliness, the results of past studies are not congruent. In other words, some prior studies revealed that men experienced greater loneliness than women (e.g., Dykstra and de Jong Gierveld 2004), whereas other studies indicated no differences (Cramer and Neyedley 1998) or women reporting greater loneliness (e.g., Jakobsson and Hallberg 2005). For example, the results obtained by Dykstra and Fokkema (2007) showed that divorced men were more likely to experience emotional and social loneliness than women. In the same study gender differences among married individuals were less consistent with married men being more likely to experience social loneliness compared to married women, but no differences emerged in regard to emotional loneliness. In other studies, male university students had higher levels of romantic loneliness, while there were no significant gender differences for either social or family loneliness (DiTommaso, Brannen-McNulty, Ross, and Burgess 2003). Furthermore, in a study by DiTommaso, Brannen, and Burgess (2005), men reported higher levels of family and social loneliness than did women. In turn, DiTommaso et al. (2007), in a study utilizing a sample of individuals aged 17 to 79 years, did not find significant gender differences in the area of three distinct domains of loneliness.

At the same time, it is important to note that the developmental tasks of women are traditionally defined in reference to relations with other people (Mandal 2004), and the need for establishing close relationships is not as strong in men as in women (Mandal 2004). In general, women are found to display a stronger desire for intimacy and higher motivation for it than men who are often described as focused on instrumentality and achievement (Feldman et al. 1998). Regarding these notions and, as suggested in the literature, since being never married may be related to different consequences for men and women (Keith 2003), it is plausible to assume, at least in regard to romantic loneliness, that the lack of a romantic partner may be related to greater romantic loneliness in single women than in single men.

### Perceived Social Support

Perceived social support refers to perceptions of the extent to which people from one’s social network are available to provide social support (e.g., Demaray and Malecki 2002) and it may have a more significant effect than actual received social support (Pinquart and Sörensen 2003; Wethington and Kessler 1986). Studies have demonstrated that social support is an important variable in lowering loneliness (e.g., Bernardon et al. 2011; Deniz et al. 2005; Kara and Mirici 2004). For example, perceived friendship support was found to be the best predictor of lower loneliness scores (Pierce et al. 1991) as well as perceived social support from family and friends were found to be a buffer against loneliness in the study by Schmitt and Kurdek (1985).

In regard to relationship status, current involvement in a romantic relationship was found to be a factor differentiating the level of perceived social support. In a study by Zimet,

Zimet, Powell, Farley, Werkman, and Berkoff (1990), compared to single individuals, married individuals reported significantly greater support from the significant other. Similarly, Prezza and Pacilli (2002) found that married people declared higher support from the significant other than unmarried people, but there was no difference in family support between married and single participants. In a Polish study conducted on a sample of university students aged 19 to 25 years, the single participants reported less subjectively perceived social support from significant others than the partnered participants, but no significant differences between the both groups existed in regard to family and friends support (author citation).

Concerning gender differences in the domain of social support, previous research provided inconsistent findings with several studies indicating no gender differences and others indicating higher levels of support among women (Coventry et al. 2004). Women were found to have larger overall social support networks than men and to turn to various sources of support more often than men (Day and Livingstone 2003). At the same time, gender differences were found in regard to the use of specific sources of support. In particular, women have tendency to turn for support to friends, co-workers and family, whereas men were found to report higher perceived support from their bosses than did women (Day and Livingstone 2003). Furthermore, in the study by Zimet et al. (1988), women reported greater support from the significant other and friends, and greater overall support than men, but no gender differences in terms of support from family. In a Turkish study Duru (2007) found that female students reported receiving greater support from family and friends, and greater total support than male students. In the study ran on a sample of undergraduate psychology students aged 17 to 52 years (*M* = 21.50, *SD* = 5.0). Day and Livingstone (2003) found that women indicated that they would turn to their partner and friends more often than men would. Prezza and Pacilli (2002), in their study utilizing a sample of individuals aged 18 to 77 years, found that men received more support from the family, particularly when very young (from 19 to 25 years of age) or older (from 46 to over 65 years of age). Younger women (up to about the age of 45) had higher support from friends than their male counterparts.

At the same time, considering that at least marriage is believed to represent more social, psychological, and economic benefits for men than for women (Bernard 1972), that in general women are found to have better support networks than men (e.g., Turner and Marino 1994), and to receive more social support from friends and family in their larger social networks (Carbery and Buhrmester 1998), it is plausible to assume that single women may experience greater perceived social support than single men.

### The Aims and Hypotheses of the Study

The present investigation was performed as a cross-sectional study in which correlational associations between relationship status, gender, loneliness and perceived social support were analyzed. The purpose of the present study was twofold. First, employing the multidimensionality approach to loneliness and perceived social support, this study aimed to verify the common beliefs about loneliness and social isolation of singles by comparing single young adults with young adults in nonmarital romantic relationships. The focus on young adults derives from the following reasons: (1) the engagement in a stable intimate relationship is one of the most prominent social roles in late adolescence and young adulthood (Roberts and Wood 2006); (2) individuals aged 18 to 24 years experience significant levels of loneliness (Eshbaugh 2010), and the transition to adulthood may be associated with feelings of loneliness in one domain and not another, depending on what particular relationship deficit is experienced by an individual (Bernardon et al. 2011). In addition, to date, most research has focused on comparisons between single individuals with their married counterparts. This study compares single individuals with individuals in nonmarital romantic relationships as these relationships play a crucial role in young adults’ lives, their identity, self-concept, and psychological well-being (Simon and Barrett 2010). Furthermore, contrary to most studies which focused on loneliness conceptualized from a unidimensional perspective, the present study focuses on family, social, and romantic loneliness and perceived social support from friends, family and significant others, since social support was found to be a significant factor contributing to the reduction of loneliness among adolescents, university students, adults, and elderly people (Kapıkıran 2013). It is important to note that the multidimensional approach might be especially useful for studying loneliness among young adults who undertake developmental tasks, enter into new social networks and new social roles, including a role of a lifetime partner/spouse, and find new sources of social support (Bernardon et al. 2011). Moreover, an investigation of distinctive sources of perceived social support enables an assessment of the significance of particular sources of social support in the situation of being single *vs.* being in a nonmarital relationship. In addition, the current study also focuses on gender differences in the domain of loneliness and perceived social support since gender is considered to be an important factor in the sphere of romantic relationships (e.g., Brannon 2002; Mandal 2004).

The second aim of the study was to determine the link between the duration of remaining single and single young adults’ romantic loneliness as a result of the lack of a romantic partner, with special attention paid to perceived social support from family, friends and significant others as a moderator of this relationship. In particular, the study explored the potential interaction effects of perceived social support on single young adults’ romantic loneliness in order to determine whether various levels of the three different sources of perceived support can prevent romantic loneliness among individuals who are single for different periods of time.

In light of the research presented in the theoretical section demonstrating differences between single and partnered individuals in regard to loneliness and perceived social support, gender differences in the domain of loneliness and perceived social support, and the buffering role of perceived social support for loneliness, the following hypotheses were formulated:

- H1: Single individuals would report a higher level of romantic loneliness in comparison to individuals in nonmarital romantic relationships.
- H2. Single individuals would report similar levels of family and social loneliness as individuals in nonmarital romantic relationships.
- H3: Single individuals would report a lower level of perceived social support from significant others in comparison to individuals in nonmarital romantic relationships.
- H4. Single individuals would report similar levels of perceived social support from family and friends as individuals in nonmarital romantic relationships.
- H5. There are gender differences in the domain of emotional (romantic and family) and social loneliness.
- H6. Relationship status will interact with gender such that single women would report greater romantic loneliness than would single men.
- H7. There are gender differences in the domain of perceived social support.
- H8. Relationship status will interact with gender such that single women would report greater perceived social support than would single men.
- H9. A longer duration of remaining single predicts greater single young adults’ romantic loneliness.
- H10. All else being equal, perceived social support from significant others, family and friends will interact with the duration of remaining single such that individuals who have been single for a long time but have high levels of perceived social support will experience lower romantic loneliness.

## Method

### Participants and Procedure

The study was carried out on a sample of university students from different departments at Adam Mickiewicz University in Poznań, Poland and included three hundred and fifteen participants (167 women and 148 men). Five hundred questionnaires were originally distributed. A total of 405 participants returned questionnaires (response rate = a 81 %). Of these, 90 participants were removed because they were married or because of incomplete data, yielding a final sample of 315 participants.

Participants were aged 19 to 25 years old (*M* = 21.90, *SD* = 2.15), resident in a large Polish city with a population exceeding 500.000 inhabitants. All the respondents reported being heterosexual, unmarried, and childless. One hundred and thirty nine students (44 %) reported being in a romantic relationship at the time of the assessment, while 176 students (56 %) were not. Being single was defined as “not in a committed relationship for at least 6 or more months, but wanting to become committed in the near future (within the next year or so)”, and being in a nonmarital romantic relationship was defined as “in a committed nonmarital relationship for at least 6 or more months, and wanting to be committed in the near future (within the next year or so)” (see Schachner et al. 2008). The mean duration of remaining single was 7.66 years with the standard deviation of 8.99 years, whereas the mean duration of being in a nonmarital romantic relationship was 3.45 years with the standard deviation of 3.03 years.

The questionnaire packages were administered in classrooms to groups of 30 to 50 students at a time and participation was voluntary. An explanation as to the purpose of the study was given along with assurance to students that all information provided would remain anonymous and confidential. The instructions were read aloud. Participants completed a demographic questionnaire and a package of two measures. The presented study was performed in line with the ethical guidelines included in the Polish Code of Professional Ethics for the Psychologist which apply to psychologists who are researchers and practitioners.

### Materials

The questionnaire package presented to the study participants was comprised of the following instruments:

#### Demographic Questionnaire

This questionnaire was designed to obtain general descriptive information about participants’ background such as their age, gender, field of study, current relationship status, and the duration of remaining single *vs.* duration of being in a romantic relationship. To determine the current relationship status, participants were asked to answer “Yes” or “No” to the question whether they have a romantic partner. In turn, to determine the duration of remaining single *vs.* duration of being in a romantic relationship, participants were asked to provide the number of years or months of how long they had remained single or how long they had been in a given nonmarital romantic relationship.

#### The social and Emotional Loneliness Scale for Adults - Short Form

(SELSA-S; DiTommaso et al. 2004) (Polish adaptation - Adamczyk and DiTommaso 2014). The SELSA-S is a multidimensional measure of loneliness which consists of 15 items rated on a 7-point Likert-type scale, ranging from *1* (strongly disagree) to *7* (strongly agree). It was designed to measure emotional (romantic and family) and social loneliness. Each subscale consists of five statements about feelings of loneliness within the past year. The family loneliness subscale assesses feelings toward family relationships. The social loneliness subscale measures feelings toward being part of a social group. The romantic loneliness subscale measures the degree to which participants feel they have significant others in their lives. Mean scores are calculated for each subscale, and higher SELSA-S scores indicate higher levels of loneliness in the particular domain. The SELSA-S’s three subscales have high internal reliability, with Cronbach’s alpha coefficients ranging from 0.87 to 0.90, and have been shown to be a valid measure of loneliness (Çeçen 2007; DiTommaso et al. 2004; DiTommaso et al. 2007). In the present study, Cronbach’s alpha coefficients were as follow: 0.83 (romantic), 0.87 (family), and 0.84 (social).

#### The Multidimensional Scale of Perceived Social Support

(MSPSS; Zimet et al. 1988) (Polish adaptation - Adamczyk 2013). This scale is a 12-item self-report instrument designed to assess a person’s perception of the adequacy of social support from three distinct sources: friends, family, and significant others. The friends support subscale measures subjective perceptions of social support from friends. The family support subscale measures subjective perceptions of social support from family, and the significant other support subscale measures subjective perceptions of social support from friends significant others in participants’ lives. There are four items per subscale, each with response options ranging from 1 (*very strongly disagree*) to 7 (*very strongly agree*). Higher scores on each of the subscales indicate higher levels of perceived support. The three subscales have high internal reliability. Specifically, the Cronbach’s alpha coefficients of the subscales were *α* = 0.85 (for friends), *α* = 0.87 (for family) and *α* = 0.91 (for significant other). The MSPSS has been shown to be a valid measure of perceived social support (Zimet et al. 1988; Zimet et al. 1990). In the present study, the following Cronbach’s alpha coefficients were obtained for the MSPSS subscales: 0.90 (significant other), 0.99 (family), . 94 (friends), and for the total scale 0.89.

## Results

### Data Analysis

In the first step, in order to verify the hypotheses concerning possible differences between single individuals and individuals in nonmarital romantic relationships, and between women and men in regard to loneliness and perceived social support, as well as in order to examine possible interactional effects of relationship status and gender on loneliness and perceived social support, a two-way multivariate analysis of variance (MANOVA) was performed. In the next step, in order to investigate the predictive role of the duration of remaining single and the moderating role of perceived social support from family, friends and significant others for single individuals’ romantic loneliness, a hierarchical regression analysis was performed for the sample of single individuals (*n* = 176). For clarity of presentation, the results are presented separately in five major sections. The first and second sections delineate results concerning differences between single individuals and individuals in nonmarital romantic relationships in regard to loneliness and perceived social support. The third and fourth sections present findings on gender differences and the interactional effect of relationship status and gender in the domain of loneliness and perceived social support. Finally, the last section is devoted to the presentation of results concerning the predictive role of the duration of remaining single and the moderating role of perceived social support from family, friends and significant others for single individuals’ romantic loneliness.

### Relationship Status and Loneliness

In order to examine the possible mean differences between single individuals and individuals in nonmarital romantic relationships in regard to loneliness a multivariate analysis of variance (MANOVA) was used. The analysis resulted in a significant multivariate effect of relationship status on loneliness, Wilks’s Λ = 0.49, *F*(3, 309) = 106.79, *p* < 0.001, *η* ^*2*^ = 0.51.

Follow-up univariate analyses revealed that single individuals scored higher on romantic loneliness than did individuals in nonmarital romantic relationships (see Table 1). Furthermore, single individuals scored higher on family loneliness than did individuals in nonmarital romantic relationships. At the same time, no differences emerged between single individuals and individuals in nonmarital romantic relationships in the domain of social loneliness.

**Table 1:** Means and standard deviations on loneliness and perceived social support by relationship status

Variable	Total sample (*N* = 315)	Single sample (*N* = 176)	Partnered sample (*N* = 139)	*F* ratio	*η2*
	Mean (SD)	Mean (SD)	Mean (SD)		
Multivariate test				106.79***	0.51
Loneliness					
Romantic loneliness	15.60 (8.51)	21.17 (6.38)	8.55 (4.86)	306.93***	0.50
Family loneliness	11.14 (5.74)	11.78 (6.18)	10.34 (5.03)	6.45*	0.02
Social loneliness	11.17 (5.25)	11.17 (5.55)	11.19 (4.86)	0.92	0.00
Multivariate test				44.90***	0.30
Perceived social support					
Significant other support	18.66 (4.14)	17.07 (4.52)	20.69 (2.38)	118.01***	0.28
Family support	17.19 (4.29)	16.95 (4.51)	17.48 (3.99)	4.88*	0.02
Friends support	17.67 (3.89)	17.80 (4.01)	17.51 (3.74)	1.94	0.01

***p < .001; *p < .05

### Relationship Status and Perceived Social Support

In order to test the hypothesis that single individuals and individuals in nonmarital romantic relationships will differ in regard to perceived social support a multivariate analysis of variance was applied. The analysis disclosed a significant multivariate effect of relationship status on perceived social support, Wilks’s Λ = 0.70, *F*(3, 309) = 44.90, *p* < 0.001, *η* ^*2*^ = 0.30.

Follow-up univariate analyses (see Table 1) revealed that single individuals scored lower on perceived social support from significant others compared to individuals in nonmarital romantic relationships. In addition, single individuals scored lower on perceived social support from family compared to individuals in nonmarital romantic relationships, but no differences emerged between the two groups in regard to perceived social support from friends.

### Gender and Loneliness

In order to explore the possible gender differences in the domain of loneliness and possible interactional effect of relationship status and gender on loneliness, a two-way MANOVA was performed. The analysis showed a significant multivariate effect of gender on loneliness, Wilks’s Λ = 0.96, *F*(3, 309) = 4.19, *p* < 0.01, *η* ^*2*^ = 0.04. At the same time, the interactional effect of relationship status and gender emerged to be insignificant, Wilks’s Λ = 0.99, *F*(3, 309) = 0.90, *p* > 0.05, *η* ^*2*^ = 0.00.

As shown in Table 2, men scored higher on social loneliness than women, but no gender differences emerged in the extent of romantic and family loneliness.

**Table 2:** Means and standard deviations on loneliness and perceived social support by gender

Variable	Total sample (*N* = 315)	Women (*N* = 167)	Men (*N* = 148)	*F* ratio	*η2*
	Mean (SD)	Mean (SD)	Mean (SD)		
Multivariate test				4.19**	0.04
Loneliness					
Romantic loneliness	15.60 (8.51)	18.12 (8.13)	12.76 (8.05)	2.28	0.00
Family loneliness	11.14 (5.74)	11.01 (6.01)	11.30 (5.43)	1.65	0.00
Social loneliness	11.17 (5.25)	10.43 (5.17)	12.01 (5.23)	7.25**	0.02
Multivariate test					
Perceived social support				15.06***	0.13
Significant other	18.66 (4.14)	19.14 (3.62)	18.14 (4.60)	33.76***	0.10
Family support	17.19 (4.29)	17.80 (4.17)	16.50 (4.34)	10.72***	0.03
Friends support	17.67 (3.89)	18.78 (3.38)	16.42 (4.05)	30.98***	0.09

***p < .001; ** *p* < .01

### Gender and Perceived Social Support

In order to examine the possible mean differences between women and men in regard to perceived social support as well as in order to investigate the possible interactional effect of relationship status and gender on perceived social support, a two-way MANOVA was performed. The analysis showed a significant multivariate effect of gender on perceived social support, Wilks’s Λ = 0.87, *F*(3, 309) = 15.06, *p* < 0.001, *η* ^*2*^ = 0.13. As shown in Table 2, women scored higher on all three distinct sources of perceived social support.

At the same time, the interactional effect of relationship status and gender emerged as significant, Wilks’s Λ = 0.93, *F*(3, 309) = 7.49, *p* < 0.001, *η* ^*2*^ = 0.07. The interactional effect of relationship status and gender emerged for perceived social support from significant others, *F*(1, 311) = 20.63, *p* < 0.001, *η* ^*2*^ = 0.06 and for perceived social support from friends, *F*(1, 311) = 4.45, *p* < 0.05, *η* ^*2*^ = 0.01.

In order to understand these interactional effects, an analysis of the simple main effects of relationships status in the group of women and men was performed. With respect to perceived social support from significant others, the analysis revealed a significant simple main effect of relationship status in the group of women, *F*(1, 311) = 19.63, *p* < 0.001, partial η^2^ = 0.06, and men, *F*(1, 311) = 120.85, *p* < 0.001, partial η^2^ = 0.28. To be precise, single status was associated with lower perceived social support from significant others in the group of women (*M* = 18.41) and men (*M* = 14.11). At the same time, the mean difference between the mean level of perceived social support from significant others reported by single and partnered individuals was bigger for the group of men, and the lowest level of perceived social support from significant others was reported by single men.

With respect to perceived social support from friends, the analysis revealed a significant simple main effect of relationship status in the group of men, *F*(1, 311) = 6.25, *p* < 0.05, partial η^2^ = 0.02, but not in the group of women, *F*(1, 311) = 0.25, *p* > 0.05, partial η^2^ = 0.00. In other words, single men reported significantly lower perceived social support from friends (*M* = 15.44) than men who were in nonmarital romantic relationships (*M* = 17.00). This relationship was not observed in the group of women, where single women reported similar level of perceived social support from friends (*M* = 18.87) as women in nonmarital romantic relationships (*M* = 18.55).

### The Predictive role of the Duration of Remaining Single and the Moderating Effect of Perceived Social Support for the Linkage Between Duration of Remaining Single and Single Young Adults’ Romantic Loneliness

In order to investigate the predictive role of the duration of remaining single and the moderating role of perceived social support from family, friends and significant others for romantic loneliness in the sample of single individuals (*n* = 176), a hierarchical regression analysis was performed. The interaction terms that were used were created as the products of the duration of remaining single and perceived social support types, with both variables first converted to *z*-scores. In the first step, the duration of remaining single and three distinct sources of perceived social support were examined. In the second step, the interaction between the duration of remaining single and three distinct types of perceived social support was investigated. Table 3 presents the standardized betas, adjusted *R* ^*2*^-values, and *R* ^*2*^-change values for the subsequent steps in the regression analysis.

**Table 3:** Hierarchical regression analysis predicting romantic loneliness from duration of remaining single and perceived social support from family, friends and significant others

Predictor	Romantic loneliness	*R* ^*2*^	*ΔR* ^*2*^
	*β*		
Step 1		0.12	0.14***
Duration of remaining single	0.30***		
Family social support	0.06		
Friends social support	0.05		
Significant others social support	−0.24*		
Step 2		0.16	0.05*
Duration of remaining single	0.40***		
Family social support	0.06		
Friends social support	0.07		
Significant others social support	−0.25*		
Interaction Duration of remaining single * Family social support	−0.20*		
Interaction Duration of remaining single * Friends social support	−0.15		
Interaction Duration of remaining single * Significant others social support	0.23*		

*p < .05; ** *p* < .01; *** *p* < .001

As Table 3 demonstrates, in Step 1 the main effects of the duration of remaining single and perceived social support from significant others explained a significant portion of the variance in the outcome. In particular, the duration of remaining single was positively and moderately related to romantic loneliness, whereas perceived social support from significant others was negatively and weakly related to romantic loneliness. In Step 2, the duration of remaining single and interaction effects of the duration of remaining single and perceived social support from significant others and family significantly added to the prediction of romantic loneliness.

**Fig. 1:**
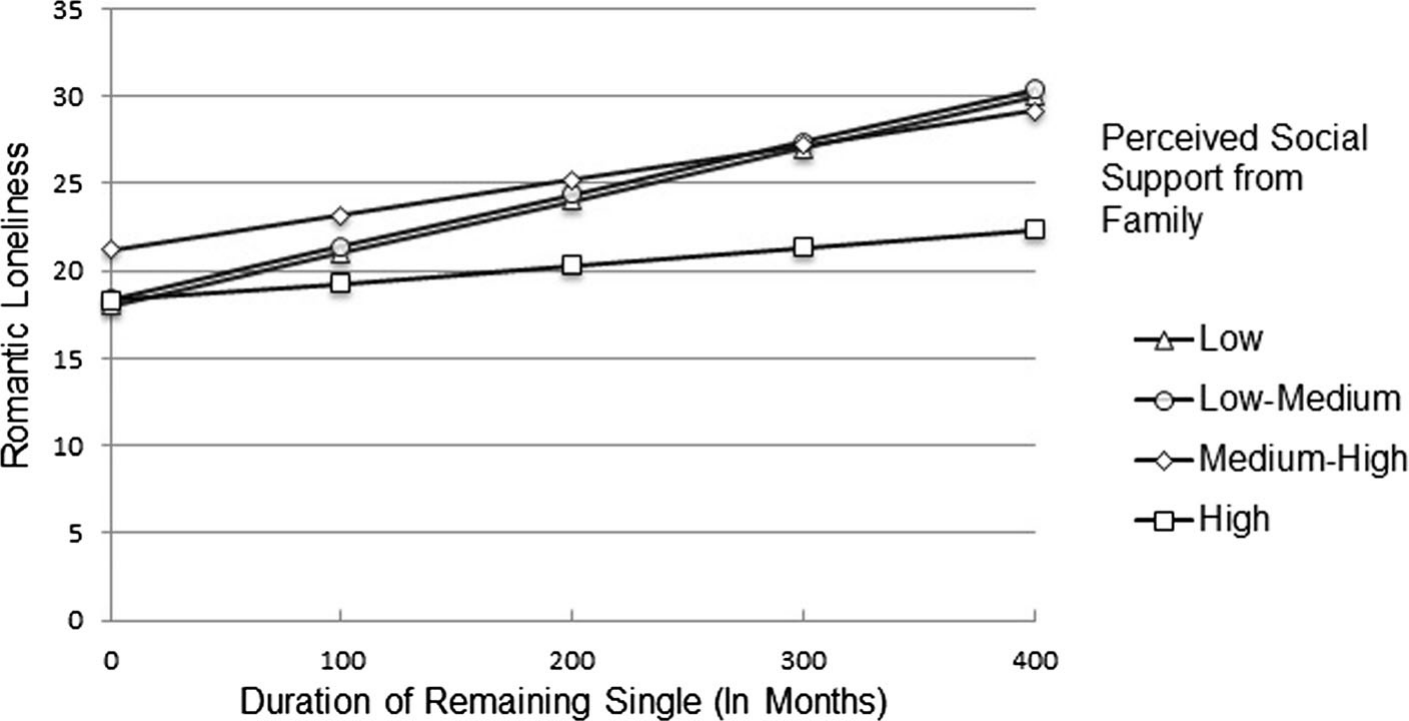
Interaction effect of perceived social support from family x duration of remaining single for romantic loneliness

In order to determine the interactional effect of perceived social support from family, this variable was categorized by visual binning on the basis of cut-points at the mean and +/- one standard deviation. As a result, the following four categories of perceived social support from family were obtained: low (<= 12.47), low-medium (12.48–16.98), medium-high (16.99–21.48), and high (21.49+). The analysis of the duration of remaining single and romantic loneliness in the above groups indicated that this relationship was positive and moderate in the following groups of perceived social support from family: low family support, *β* = 0.52, *p* < 0.01, low-medium family support, *β* = 0.47, *p* < 0.001, and in the group of medium-high family support, *β* = 0.33, *p* < 0.05. In the group of high family social support this relationship was insignificant, *β* = . 20, *p* > 0.05. Figure 1 demonstrates that the relation between romantic loneliness and the duration of being single changes as a function of the level of perceived social support from family.

Figure 1 demonstrates that as perceived social support from family increases to a high level, the relationship between romantic loneliness and the duration of being single is no longer significant. In other words, the higher the reported perceived social support from family, the more it mitigates the negative impact on romantic loneliness of being single for a long period of time.

**Fig. 2:**
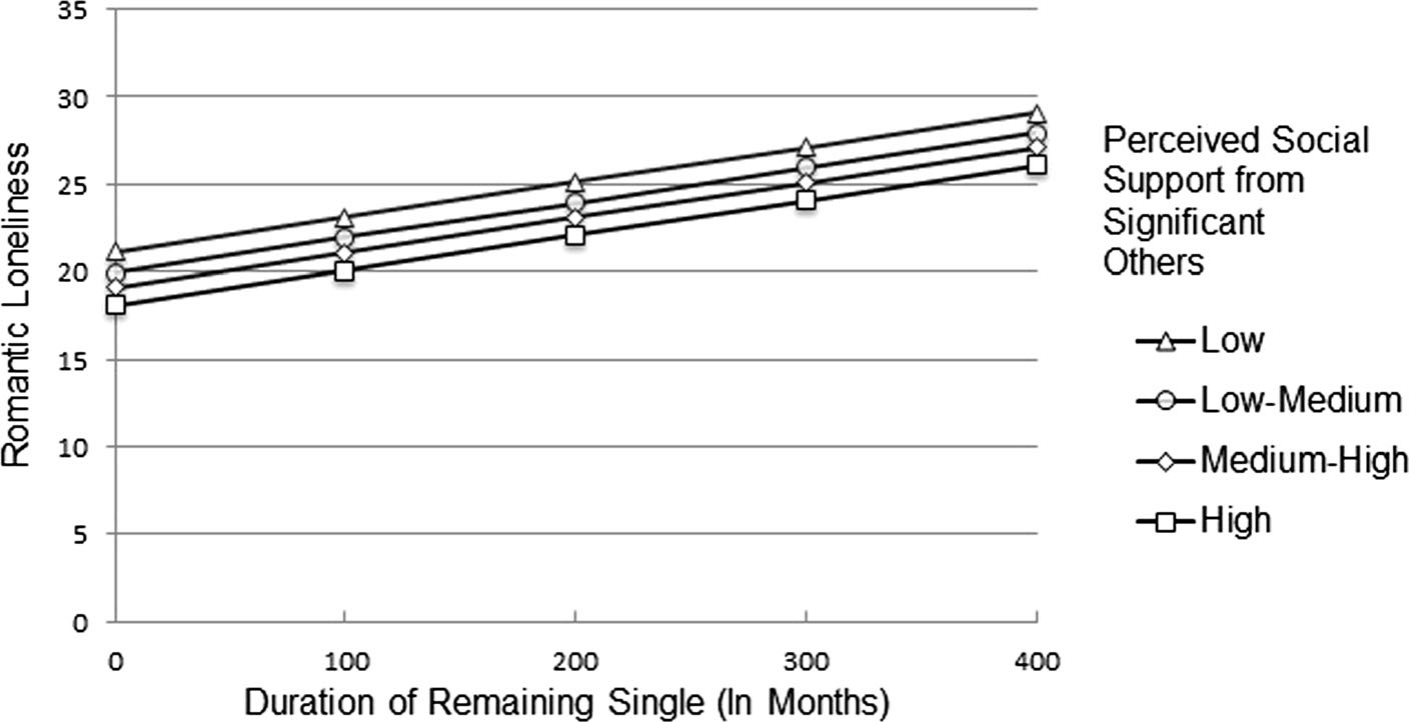
Interaction effect of perceived social support from significant others x duration of remaining single for romantic loneliness

In order to determine the interactional effect of perceived social support from significant others, this variable was categorized by visual binning on the basis of cut-points at the mean and +/- one standard deviation. As a result, the following four categories of perceived social support from significant others were obtained: low (<= 12.57), low-medium (12.58–17.07), medium-high (17.08–21.58), and high (21.59+). The analysis of the duration of remaining single and romantic loneliness in these groups indicated that this relationship was positive and moderate in the following groups: low significant others support, *β* = 0.40, *p* < 0.05, low-medium significant others support, *β* = 0.31, *p* < 0.05, and in the group of high significant others support, *β* = 0.38, *p* < 0.05. In the group of medium-high significant others social support the relationship was insignificant (*β* = . 25, *p* > 0.05). Fig. 2 demonstrates that the relation between romantic loneliness and the duration of being single changes as a function of the level of perceived social support from significant others.

Figure 2 demonstrates that as perceived social support from significant others increases to a medium-high level, the relationship between romantic loneliness and the duration of being single is no longer significant. In other words, the medium-high level of perceived social support from significant others mitigates the negative impact on romantic loneliness of remaining single for a long period of time.

## Discussion

The present study involved a sample of Polish young people and was aimed at examining the possible differences between single young adults and young adults in nonmarital romantic relationships with respect to the domain of social and emotional (i.e., romantic and family) loneliness and perceived social support from family, friends and significant others. In addition, gender differences in regard to loneliness and perceived social support, and the interactional effect of relationships status and gender on loneliness and perceived social support were investigated. The present study also intended to examine the predictive role of remaining single for single young adults’ romantic loneliness and the role of perceived social support as a moderator of this relationship. In general, the present study showed that, in comparison to individuals in nonmarital romantic relationships, single persons experience loneliness in specific domains (i.e., in the domain of romantic partners and family), but not in other domains (i.e., in the domain of social relationships). Similarly, in regard to perceived social support the present study indicated that single individuals have a lower perception of selected sources of social support (i.e., family and significant others support), but, at the same time, they may have similar subjectively perceived support from friends as individuals in relationships. These findings contradict the perception of singles as completely lonely and having no friends (Greitemeyer 2009; DePaulo and Morris 2005).

### Relationship Status and Loneliness

In line with the first hypothesis, it was expected that single individuals would report a higher level of romantic loneliness in comparison to individuals in nonmarital romantic relationships. The present study showed that, in comparison to individuals in nonmarital romantic relationships, single participants scored higher on romantic loneliness. This pattern of results confirms the importance of having a romantic partner in young adulthood when young adults are expected to undertake new social roles such as being a partner or a spouse, and to establish a stable relationship (Lodi-Smith and Roberts 2007). In result, the lack of a romantic partner maybe related to loneliness (e.g., Deniz et al. 2005; Green et al. 2001). Similarly, married individuals are found, on average, to be less lonely than unmarried individuals (Tornstam 1992), and living with a partner predicts the lowest levels of loneliness (de Jong-Gierveld 1987).

The second hypothesis assumed that single individuals would report similar levels of family and social loneliness as individuals in nonmarital romantic relationships. This hypothesis was only partially supported. The obtained results revealed that single individuals experienced higher level of family loneliness than individuals in nonmarital romantic relationships, but also that they experienced comparable level of social loneliness as their peers in nonmarital romantic relationships. The findings may be understood if we take into consideration that during adulthood romantic partners take a special position in the network of attachment figures and become a primary attachment figure (Rowe and Carnelley 2005). Thus, it is plausible to assume that young adults who are in a committed relationship may perceive their romantic partners as family members, and as significant others with whom they want to start their own families (DiTommaso et al. 2007). At the same time, young adults who are single may have a higher level of familys loneliness, making unfavourable comparisons with their peers in nonmarital romantic relationships who are achieving important developmental tasks in the area of marital and family life with their romantic partners. The comparable level of social loneliness among single individuals and individuals in nonmarital romantic relationships can be related to the notion that social loneliness refers primarily to unmet needs in the wider network of support givers (Dykstra and Fokkema 2007). Social loneliness as a result of an inadequate access to social relationships such as a network of peers, co-workers, neighbours, or friends (Weiss 1973), is not associated with the lack of a romantic partner. This would explain similar level of social loneliness among single individuals and individuals in romantic relationships.

### Relationship Status and Perceived Social Support

According to the third hypothesis, it was expected that single individuals would report a lower level of perceived social support from significant others in comparison to individuals in nonmarital romantic relationships. The present study supported this assumption showing that single individuals reported less perceived social support from significant others in comparison to individuals in nonmarital romantic relationships. This pattern of results is consistent with prior studies indicating, for example that married individuals report significantly greater support from the significant other than single individuals (Prezza and Pacilli 2002; Zimet et al. 1990). Analogically, in the study performed by Zimet et al. (1990) married residents reported significantly greater support from a significant other than single residents, whereas no significant differences were found for the family and friends subscales. The present results showed that relationship status in young adulthood can be an important determinant of perceived support, just as marital status was found to be one among the elderly (Cutrona 1986).

The fourth hypothesis assumed that single individuals and individuals in nonmarital romantic relationships would not differ in terms of their perception of social support from family and friends. This hypothesis was only partially supported. The present study demonstrated that single individuals and individuals in nonmarital romantic relationships reported comparable level of perceived social support from friends. At the same time, contrary to the hypothesis, single individuals reported less perceived social support from family compared with individuals in nonmarital romantic relationships. The lower level of perceived social support from family among single individuals – analogously to family loneliness among single individuals – can be related to the possible perception of romantic partners as family members. As a result, single young adults not having romantic partners treated as their family member may, in comparison to their peers in nonmarital romantic relationships, report lower perceived social support from family. In turn, with respect to similar level of perceived social support from friends among single individuals and individuals in relationships it can be indicated – similarly as in regard to the comparable level of social loneliness among single individuals and individuals in relationships – that single people have similar access as people in relationships to social relationships such as a network of peers, co-workers, neighbors or friends. Therefore, single individuals as well as individuals in relationships perceive their friends as similarly supporting.

### Gender and Loneliness

With respect to the fifth hypothesis, it was predicted that gender differences would emerge in regard to loneliness. Results revealed that men and women did not differ in the domain of romantic and family loneliness, however men reported higher levels of social loneliness than women. It is possible that this pattern of findings is associated with changes observed in recent decades and related to a diminishing pattern of gender differences in the sphere of intimacy during young adulthood (Feldman et al. 1998). These changes are thought to contribute to the acknowledgement by men of the benefits deriving from intimacy and closeness with a partner (Feldman et al. 1998). Furthermore, in a more recent study by Perrin et al. (2011) behaviors which women and men desired and received from romantic relationships were much more alike than different. Thus, as gender differences in the domain of romantic relationships appear to diminish, it is possible that men and women have similar experiences in the domain of romantic and also family relationships, which also require loving behaviors.

With respect to higher levels of social loneliness in men, the present findings corroborate the findings of the study by Dykstra and Fokkema (2007). In these authors’ study, men – regardless of partner status – had smaller support networks and higher levels of social loneliness in comparison to women. The obtained results may be related to the notion that social loneliness is more common among people with a relatively small social network (i.e., relatives, colleagues, friends, neighbors) (Dykstra and Fokkema 2007). In relation to the above, when compared to men, women have larger social networks and are more likely to indicate children, family, and friends as sources of support, while men usually indicate only or mainly their spouses (Pinquart and Sörensen 2003). As a result, men may experience higher social loneliness relative to women.

In addition, contrary to the sixth hypothesis, which predicted that relationship status would interact with gender such that single women would report greater romantic loneliness than would men, the performed analysis revealed no interaction between relationship status and gender in the domain romantic loneliness. The assumption of existence of the interactional effect of relationship status and gender on romantic loneliness was based on the literature indicating that women are believed to have a stronger interest in establishing close, dyadic social ties (Stokes and Levin 1986) and also, in line with commonplace beliefs, that men are less willing to connect with others than women (Schmitt 2008). Therefore, it was expected that the absence of a close, intimate relationship will be particularly detrimental for women, who allegedly seek a long-term mate, as opposed to men, who avoid commitment (Perrin et al. 2011), which will contribute to higher romantic loneliness among single women. The present study showed– at least in the case of young adults who are university students – that singlehood among women and men is related to similar levels of loneliness. It is plausible that this pattern of results is also related to the changes indicated in regard to loneliness, that is, changes in the sphere of intimacy during young adulthood consisting in the diminishing pattern of gender differences (Feldman et al. 1998). In addition, a possible explanation of lack of differences in the domain of romantic loneliness among women and men with respect to relationship status may be related to the suggestions made in the literature that women, despite their greater need for affiliation as compared to men, more often than men chose the single lifestyle since they value more their individual success and independence (Żurek 2005).

The choice of the single lifestyle may be also related to women’s recognition that marriage is considered to represent more social, psychological and economic benefits for men than for women (Bernard 1972). As a result, women, at least those who choose singlehood, may experience similar levels of romantic loneliness as single men.

### Gender and Perceived Social Support

In the present study it was hypothesized (H7) that gender differences would emerge in the domain of three sources of social support. The obtained results confirmed this hypothesis, indicating that women experienced higher perceived social support from three distinct sources. Prior research on gender differences in social support provided inconsistent findings indicating no gender differences or greater support in women (Coventry et al. 2004). Furthermore, prior studies indicated greater support from the significant other and friends among women (Zimet et al. 1988), greater support from family and friends, and greater total support among female students (Duru 2007) or more support from the family received by men (Prezza and Pacilli 2002). The results of the current study are in agreement with this literature showing that in general women are found to have better support than men (e.g., Turner and Marino 1994), receive more social support from friends and family and have a larger social network (Carbery and Buhrmester 1998; Dykstra and Fokkema 2007), and that they seek support from friends and families to a greater degree than men in order to cope with stressful situations (Day and Livingstone 2003).

In addition, the eighth hypothesis predicted that relationship status will interact with gender such that single women would report greater perceived social support than would single men. In the present study the analysis of the simple main effect of relationship status for perceived social support from significant others and friends revealed that (1) both in the group of women and men relationship status was related to different levels of perceived social support from significant others; that is, single status was related to a significant decrease of this support, with single men reporting the lowest level of this support compared to single and partnered individuals; and (2) only in the group of men was relationship status related to different levels of perceived social support from friends; that is, single status was related to a clear decrease of this support among men but not among women. This pattern of results may be understood by recalling that men are generally more socially isolated than women because they do not create adequate emotional intimacy when they are not in a partnership with a significant other (Vandervoort 2000). In addition, living without a partner was found to be more difficult for men than for women (Chipperfield and Havens 2001). Therefore, among single men, the lack of a life partner may be particularly associated with lower levels of perceived social support from significant others and friends support.

### The Predictive Role of the Duration of Remaining Single and the Moderating Effect of Perceived Social Support for the Linkage Between Duration of Remaining Single and Single Young Adults’ Romantic Loneliness

In line with the ninth hypothesis, it was expected that a longer duration of remaining single would predict greater single young adults’ romantic loneliness. The performed analysis provided support for this expectation, indicating that a longer duration of being single (measured in months) was related to a higher level of romantic loneliness among single young adults. In the current study loneliness was not examined from the temporal perspective, but it is plausible that in the case of prolonged periods of remaining single (lasting even up to several years) one may deal with chronic loneliness, as this type of loneliness is experienced when an individual has failed to establish satisfactory social relationships over years (Wang et al. 2008).

In addition, the results of hierarchical regression analysis revealed that lower perceived support from significant others was related to greater romantic loneliness. As previous studies showed, perceived social support is negatively related to loneliness (Kara and Mirici 2004). Furthermore, in Çeçen’s (2007) study ran on graduate and undergraduate students, higher social support was associated with lower social loneliness, and higher scores on significant others (dating, engagement, etc.) were associated with lower romantic loneliness. Eshbaugh (2010) in her study on college women found that social support from family, friends, and romantic partners was negatively related to loneliness measured as an unidimensional construct by using the UCLA Loneliness Scale. Regarding that significant other (i.e., as a person who is around when an individual is in need, with whom joys and sorrows can be shared, who is a real source of comfort and care of an individual’s feelings, see the MSPSS items), lower support from significant other may contribute to greater romantic loneliness among single individuals.

Finally, the tenth hypothesis predicted that perceived social support from significant others, family and friends will interact with the duration of remaining single such that individuals who have been single for a long time but have high levels of perceived social support will experience lower romantic loneliness. This hypothesis was partially supported.

The current study revealed the significance of interactions of perceived social support from family and significant others and the duration of remaining single for single individuals’ romantic loneliness. First, these results revealed that despite a prolonged period of remaining single, the level of romantic loneliness may decrease when single individuals perceive their families as highly supportive. As research on adult attachment showed, young adulthood is a period in life when romantic partners become primary attachment figures, and in the case of the lack of a lifetime partner – as it happens among single individuals – parents and siblings may still be the main attachment figures, taking the central position in the networks of attachment (Doherty and Feeney 2004), and serve as an essential source of support in the situation of the lack of a romantic partner. Family support may therefore replace social support usually provided by the partner, protecting single adults from the feelings of romantic loneliness.

In regard to perceived social support from significant others, the analysis indicated that – contrary to perceived social support from family – not a high but medium-high level of this support mitigated the negative impact on romantic loneliness of long periods of being single. Contrary to perceived social support from family, a high level of perceived social support from significant others did not buffer the negative impact on romantic loneliness of long periods of remaining single. In particular, actually both high as well as low perceived social support from significant others was related to increased romantic loneliness. In an attempt to interpret this pattern of results, the following explanation can be proposed: Family relationships are to a greater degree obligatory in comparison to other relations, for example with friends, which are more transient and voluntary (Coventry et al. 2004). In accordance with this notion, in a study by Coventry et al. (2004), the stability of perceived support from family relations such as relations with a spouse, children and parents was higher than the stability of this type of support from relatives, friends and confidants. In addition, parental support is related to the improvement of adolescents’ and young adults’ well-being (Feinstein et al. 2014). Moreover, acceptance, empathy and support provided by parents during the transition to young adulthood represent an essential factor contributing to healthy adjustment in this life period (Holahan et al. 1994). Concerning the specificity of family relationships and their role for adjustment during young adulthood, it can be concluded that support from significant others operates differently than family support. This type of support, being related to more voluntary and transient nature of relationships, may make single individuals particularly vulnerable to the character and amount of support. As prior studies indicated, at least in regard to received support, in some situations this support may be detrimental or ineffective (Bolger and Amarel 2007). These negative effects of social support among individuals receiving it can be related to the feelings of being dependent or overbenefitted, and can thus increase distress (Bolger and Amarel 2007). Social support can be conceptualized as a continuum - from weak to strong or from low through optimal to high support (Kacperczyk 2006). For example, in a study by Jaworska-Obłój and Skuza (1986), poor health of employees was related to too low or too high social support, whereas good health was related to optimally high support. This relationship can be explained by the notion that low support contributes to social isolation, whereas high support is related to excessive control over an individual by society (Kacperczyk 2006). As a result, in the situation of remaining single, low and high perceived social support from significant others with whom an individual may have voluntary relationships contributes to the feeling of being isolated (low support) or the feeling that other people (significant others but not the romantic partner) are too much engaged in a single individual’s life, which deepens their romantic loneliness.

### Limitations and Directions for Future Research

This study’s contributions to the existing literature must be considered in light of its limitations. The primary limitation is its cross-sectional design which prevents formulation of any causal relationships between relationship status, loneliness and perceived social support. For example, the social selective perspective attempts to explain the relationship between marriage and well-being by indicating that some people with problematic attributes (e.g., poor mental and physical health) are less likely to get married, and if they get married, to stay in a marital relationship (Gove et al. 1990). Regarding this explanation, it cannot be excluded that people reporting higher levels of loneliness in different domains, and lower levels of perceived social support from different sources, may be more likely to be single individuals. Thus, longitudinal research is needed to provide better insight into the linkage between young adults’ relationship status, loneliness and perceived social support.

A further limitation of the study is the specificity of the sample. All participant data were from never married, heterosexual and childless university students residing in a large city. In addition, the age of the sample utilized in the study, even though presenting unique developmental issues of this developmental stage, precludes any generalizations made in regard to individuals in middle and late adulthood. In future studies it would be useful to include non-student samples and to compared young adults with individuals in middle and late adulthood. It is likely that younger and older adults differ in their relationship needs and perceptions, as well as in their use of social support and feelings of loneliness (e.g., Bernardon et al. 2011; Gierveld and Dykstra 2008). Furthermore, it cannot be excluded that levels of loneliness and perceived social support may differ among various subcategories of singles taking into account different causes of the lack of a life partner (e.g., among divorced and widowed individuals in comparison to the never-married adults studied in the present study) (e.g., Dykstra and Fokkema 2007). Moreover, it is also probable that the pattern of results would be different for young adults living in rural areas. However, Poland is a developing country and rapid social changes, including increasing anonymity and weakening social bonds, are particularly evident in large university cities. Rural districts are still characterized by strong social ties with neighbours and relatives, and the attachment to a traditional life path (e.g., having a spouse) is more present than in Polish cities (Wojciechowski 2004). Lastly, in the current study loneliness was not measured from the temporal perspective and also no measures of objective characteristics of people’s social networks (e.g., network size and frequency of contact with network members) were included. Therefore, another suggestion for future research is to investigate loneliness from the temporal perspective in regard to the duration of remaining single and to investigate objective characteristics of people’s social networks. Alongside this suggestion, it is also recommended to include in the future studies the variable “voluntary singlehood” in order to recognize the role of one’ own choice of being single on romantic loneliness among single individuals. This recommendation is supported by a recent study (author Citation) demonstrating that individuals who perceive their singlehood as voluntary score significantly lower on romantic loneliness than individuals who perceive their singlehood as involuntary.

Despite these limitations, the present study makes a number of contributions to the literature by furthering understanding of the link between relationship status, loneliness and perceived social support in a non-American sample as mainly investigated in prior studies. In particular, the present study, by comparing single young adults with those in nonmarital romantic relationships, indicated the linkage between relationship status, loneliness and perceived social support in young adulthood which is nowadays characterized by diverse relationship statuses, not limited to marital status. Furthermore, as exemplified in the current study, single individuals might not experience loneliness in general, but solely in regard to particular domains (i.e., in a romantic and family domain). In addition, the current study revealed the role of different levels of perceived social support from family and significant others in mitigating the impact on romantic loneliness of the duration of being single. These results confirmed that social support may contribute to the decline of romantic loneliness. Furthermore, these findings demonstrated that certain sources of support may function differently than others (Dehle et al. 2001), and that kin may be more effective providers of some aspects of social support than nonkin (Cutrona 1986). Moreover, regarding the need to perform research in samples other than from the US in order to expand the scope of results from American research (Boski 2010), the current study is believed to increase the validity of American findings, concerning relationship status and its outcomes in young adulthood, to other cultures such as Polish culture.
